# Frequency of the L858R Mutation in Exon 21 of the Epidermal Growth Factor Receptor in Patients with Non-Small Cell Lung Cancer

**DOI:** 10.30699/ijp.2025.2057190.3438

**Published:** 2025-11-11

**Authors:** Hossein Ayatollahi, Amir Hossein Jafarian, Zohreh Emamdadi, Farideh Ranjbar, Hassan Mehrad-Majd, Batul Oudi

**Affiliations:** 1 Cancer Molecular Pathology Research Center, Mashhad University of Medical Sciences, Mashhad, Iran; 2 Department of Pathology, Faculty of Medicine, Mashhad University of Medical Sciences, Mashhad, Iran; 3 Clinical Research Development Unit, Ghaem Hospital, Mashhad University of Medical Sciences, Mashhad, Iran.

**Keywords:** non-small cell lung cancer, epidermal growth factor receptor, L858R mutation, exon 21, clinicopathological features

## Abstract

**Background & Objective:**

Epidermal growth factor receptor (EGFR) mutations are among the most common oncogenic drivers in non-small cell lung cancer (NSCLC). The L858R mutation in exon 21 of the EGFR gene is associated with responsiveness to targeted therapies in NSCLC patients. This study aimed to evaluate the frequency of L858R mutation and its correlation with clinicopathological characteristics in NSCLC patients.

**Methods:**

In this cross-sectional study, Allele-specific Polymerase Chain Reaction (ASPCR) was used to detect L858R mutation in genomic DNA obtained from 336 patients diagnosed with NSCLC. Patients were categorized into mutation-positive groups and mutation-negative subgroups. Associations between the L858R mutation, clinicopathological features, and overall survival were analyzed using appropriate statistical methods.

**Results:**

The L858R mutation was identified in 6% of patients (20 out of 336) and showed no significant association with clinicopathological features such as age, gender, tumor grade, histology subtypes, or metastasis (all P > 0.05). Survival analysis indicated an overall mortality rate of 81.8%, with no statistically significant difference in median survival between mutation-negative (11 months) and mutation-positive groups (8 months, P=0.246). Cox regression analysis identified Tumor Grade I as a b significant prognostic factor in both Univariate (HR=0.46, P=0.031) and multivariate (HR=0.46, P=0.040) models.

**Conclusion:**

The frequency of the L858R mutation in this Iranian cohort with NSCLC was lower than that reported in global studies. However, its association with metastasis and mortality indicates the potential clinical relevance of this mutation in treatment planning.

## Introduction

Lung cancer is among the most prevalent malignancies worldwide and remains the leading cause of cancer-related mortality in both men and women (1). In 2020 alone, approximately 1.8 million deaths were attributes to long cancer, underscoring the urgency of improving its diagnosis and management (2). Epidemiological data indicate that lung cancer is more common in men than women, with prevalence rates of 3.7% and 1.7%, respectively (3, 4). 

Lung cancers are broadly classified into two major types: small-cell lung cancer (SCLC) and non-small cell lung cancer (NSCLC)(5). NSCLC accounts for over 86% of lung cancer cases, with adenocarcinoma being the most common histological subtype, comprising approximately 40%, followed by squamous cell carcinoma (6-8).

The increasing incidence of lung cancer in recent years has been linked to several risk factors, including smoking, urbanization, air pollution, exposure to carcinogenic substances, alcohol consumption, and family history (9, 10). Although smoking remains a major risk factor, it appears to be a less strongly associated with adenocarcinoma compared to squamous cell carcinoma and SCLC (11, 12). A family history of lung cancer raises the risk for both smokers and non-smokers, highlighting a possible genetic predisposition (13). Moreover, the genetic landscape of lung cancer may vary across different populations, suggesting ethnic-specific patterns of somatic mutations (14).

Several somatic mutations have been implicated in NSCLC, particularly in genes such as EGFR, KRAS, BRAF, TP53, ALK, ROS1, and HER2, all of which play key roles in regulating gene expression, cell proliferation, differentiation, and apoptosis (11, 12, 15). Among these, KRAS and EGFR mutations are the most frequently observed in NSCLC (16).

The EGFR is a transmembrane glycoprotein with intrinsic tyrosine kinase activity. It comprises an extracellular ligand-binding domain, a single transmembrane helix, and an intracellular tyrosine kinase domain (17). Upon ligand binding, EGFR undergoes auto-phosphorylation, triggering a cascade of downstream signaling pathways including Ras/Raf/MAPK, JAK/STAT, and PI3K/AKT, which collectively promote cell proliferation and inhibit apoptosis (18). 

The L858R mutation involves a substitution of leucine with arginine at codon 858 in exon 21 of the EGFR gene, resulting in constitutive activation of the receptor and uncontrolled cell growth (19, 20). This mutation is particularly significant in the context of NSCLC, as EGFR is frequently overexpressed in these tumors and serves as a critical target for tyrosine kinase inhibitors (TKIs) (21-23). EGFR overexpression has been reported in 50-80% of NSCLC cases and is associated with angiogenesis and a poor prognosis, emphasizing the importance of accurate detection of EGFR mutations (24). 

Given the high morbidity and mortality associated with lung cancer, and the therapeutic implications of specific EGFR mutations, particularly the L858R point mutation in exon 21, further studies are warranted. This study aims to determine the frequency of L858R mutation in exon 21 of the EGFR gene and evaluate its association with clinicopathological features in patients with NSCLC.

## Materials and methods

### Patients and tissue samples

Between 2019 and 2023, a total of 336 patients were diagnosed with NSCLC at hospitals in Mashhad, Iran. Eligible participants were those whose diagnosis was confirmed through imaging and histopathological evaluations. Patients who had received any form of preoperative treatments, had a history of chemotherapy or radiotherapy, or were concurrently diagnosed with malignancies in other organs were excluded from the study. 

Informed consent was obtained from all participants prior to inclusion in the study. Ethical approval was granted by the organizational ethics committee of Mashhad University of Medical Sciences (approval code: IR.MUMS.MEDICAL.REC.1403.0180. The study complied with the ethical standards outlined in the Declaration of Helsinki and its subsequent amendments. To ensure patient confidentiality, all personal information were anonymized prior to analysis using unique coded identifiers. Clinical follow-up and survival data were retrieved from hospital medical records and pathology reports by authorized clinical personnel.

Formalin-fixed paraffin-embedded (FFPE) tumor samples were prepared for EGFR mutation analysis through a standardized three-step procedure: (1) DNA extraction, (2) Polymerase chain reaction (PCR) amplification using biotinylated primers, and (3) Hybridization of the amplified products to a test strip. The test strip was embedded with allele-specific oligonucleotide probes arranged in parallel lines, and the EGFR XL Strip Assay® (ViennaLab Diagnostics GmbH, Austria) was used. Biotinylated sequences were detected using streptavidin-alkaline phosphatase and chromogenic substrates. The assay was specifically designed to detect L858R mutation in exon 21 of the EGFR gene.

### Gene Mutation Detection

Genomic DNA was extracted from FFPE tumor sections using the FavorPrep™ Tissue Genomic DNA Extraction Mini Kit, according to the manufacturer's instructions. Briefly, paraffin was first dissolved in xylene. Tissues lysis was then performed under denaturing conditions using proteinase K and an anionic detergent in the presence of a DNA stabilizer. Subsequently, samples were incubated at 90°C to reverse formalin-induced cross-linking. DNA was then bound to the purification membrane, contaminants were washed off, and the pure and concentrated DNA was eluted and stored at -20°C. The concentration and purity of the extracted DNA were assessed using a NanoDrop™ spectrometer (Nanodrop Technologies, Inc.). A DNA sample was considered pure if it had an absorbance ratio between 1.2 and 2.0 at 260/280 nm.

### PCR and detection of EGFR mutations

Allele-specific PCR (AS-PCR) was performed to selectively amplify regions containing the L858R mutation using biotinylated primers. The PCR products were then subjected to gel electrophoresis to confirm amplification. The amplified sequences were hybridized to the EGFR XL Strip Assay, which contained allele-specific oligonucleotide probes arranged in a parallel array. This method allowed for high specificity in detecting the L858R point mutation selective hybridization and enzymatic color development.

### Statistical Analysis

Statistical analyses were conducted using SPSS software (version 22, IBM Corp.). Categorical variables were analyzed using Chi-square or Fisher's exact test, while continuous variables were assessed using independent t-tests.  Logistic regression was performed to assess the association between the L858R mutation and clinicopathological characteristics. Overall survival was analyzed using Kaplan-Meier survival curves, and differences between groups were assessed using the log-rank test. Cox proportional hazards regression models were used for univariate and multivariate survival analysis. Variables with a p-value less than 0.1 in univariate Cox regression analysis were included in the multivariate model to maintain model reliability, especially considering the limited sample size and number of mutation-positive cases. Important clinical variables such as smoking status, performance status, and comorbidities were not available and thus not included in the models. A two-sided p-value of less than 0.05 was considered statistically significant.

**Table 1:** Baseline characteristics of the study patients

variables		
Age (mean±S.D)		62.96±6.85
Gender	Male	212 (63.1%)
	Female	124 (36.9%)
Tumor grade	Grade1	22 (6.5%)
	Grade2	174 (51.8%)
	Grade3	140 (41.7%)
EGFR exon 19 mutation	YES	77 (22.9%)
	NO	259 (77.1%)
Tumor type	Adenocarcinoma	251 (74.7%)
	NSCC	54 (16.1%)
	SCC	31 (9.2%)
Metastasis	YES	122 (36.3%)
	NO	214 (63.7%)
Patient's life status	death	275 (81.8%)
	alive	61 (18.2%)

## Results

### Characteristics of patients

Of the 336 patients in the study, 212 (63.1%) were male and 124 (36.9%) were female, with a mean age of 62.96 ± 6.85 years. Regarding tumor grade, 22 patients (6.5%) had grade I tumors, 174 patients (51.8%) had grade II, and 140 patients (41.7%) had grade III tumors. 

Analysis of EGFR exon 21 mutation status showed that 20 patients (6%) carried the L858R mutation, whereas the remaining 316 patients (94%) were mutation-negative. In terms of tumor histology, adenocarcinoma was the most frequent subtype, observed in 251 patients (74.7%), and followed by NSCLC in 54 patients (16.1%) and squamous cell carcinoma (SCC) in 31 patients (9.2%). 

Metastatic disease was present in 122 patients (36.3%) at the time of diagnosis, while 214 patients (63.7%) were non-metastatic. Survival analysis showed that 275 patients (81.8%) had died, and 61 patients (18.2%) were alive at the time of data collection. The clinical and pathological characteristics of the study patients are summarized in Table 1. 

### Association between EGFR Mutation and Clinicopathological Characteristics

Patients were stratified based on the presence or absence of the EGFR exon 21 mutation. The distribution of EGFR exon 21 mutation in relation to clinicopathological features of patients is summarized in Table 2. The mean age of mutation-negative patients was 63.03 ± 6.80 years, compared to 61.90 ± 7.78 years in mutation-positive patients. This age difference was not statistically significant (P = 0.447).

Exon 21 mutations were slightly more prevalent in females (9/124, 7.3%) than in males (11/212, 5.2%), but this difference was not statistically significant (P = 0.439). With respect to tumor grade, the distribution of mutations was as follows: 15.0% in grade I, 40.0% in grade II, and 45.0% in grade III. No significant relationship was found between tumor grade and mutation status (P = 0.229). 

Adenocarcinoma cases had a higher prevalence of exon 21 mutations (70.0%) compared to those with NSCC (15.0%) and SCC (15.0%). However, this difference did not reach statistical significance (P = 0.613). Similarly, there was no significant association between mutation status and the presence of metastasis (P = 0.900). 

Regarding mortality, 5.5% (15 out of 275) of deceased patients and 8.2% (5 out of 61) of surviving patients were found to have the exon 21 mutation, with no statistically significant difference (P = 0.380).

### EGFR Mutation and Prognosis in NCLC Patients

**Figure 1:**
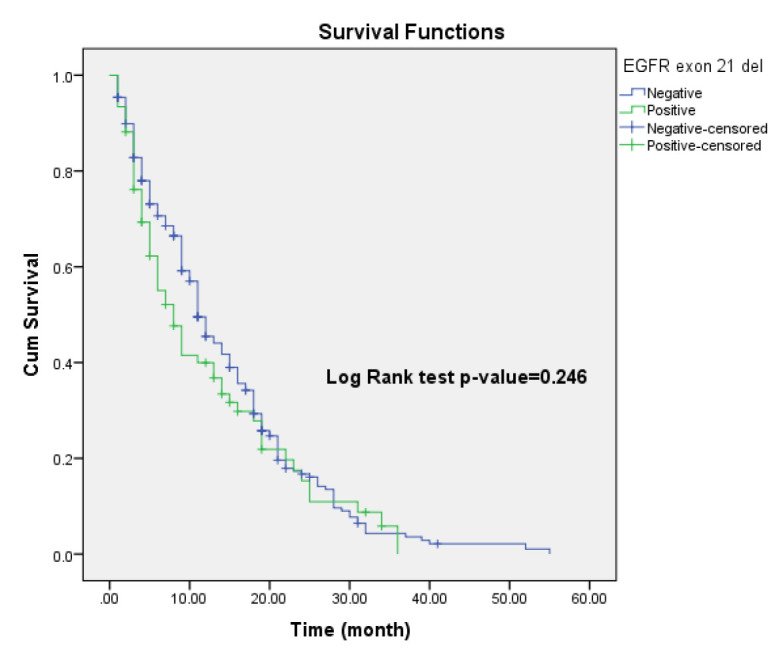
Kaplan-Meier plot to compare survival of patients according to EGFR gene mutation status

Kaplan-Meier survival analysis was used to compare survival times between mutation-negative and mutation-positive patients. The median survival time was 11 months for the mutation-negative group and 8 months for the mutation-positive group. The mean survival time was 14.19 months and 12.18 months repectively. However, this difference was not statistically significant (Log-Rank test, P = 0.246), indicating that the exon 21 L858R mutation does not significantly impact overall survival in NSCLC patients (Figure 1).

**Table 2:** Clinicopathological characteristics of patients based on the EGFR gene exon 21 mutation occurrence

variables		Exon 21 EGFR Mutation		P-Value
		Negative N=316	Positive N=20	
Age (mean±S.D)		63.02 ±6.80	61.90 ±7.78	0.477
Gender	Male	201 (63.6)	11 (55.0)	0.439
	Female	115 (36.4)	9 (45.0)	
Tumor Grade	Grade 1	19 (6.0)	3 (15.0)	0.229
	Grade 2	166 (52.5)	8 (40.0)	
	Grade 3	131 (41.5)	9 (45.0)	
Tumor type	Adenocarcinoma	237 (75.0)	14 (70.0)	0.613
	NSCC	51 (16.1)	3 (15.0)	
	SCC	28 (8.9)	3 (15.0)	
Metastasis	YES	115 (94.3%)	7 (5.7%)	0.900
	NO	201 (94.4%)	13 (5.6%)	
Patient's life status	Dead	260 (82.3)	15 (75.0)	0.380
	Live	56 (17.7)	5 (25.0)	

Cox proportional hazards regression analysis identified tumor grade as a significant prognostic factor. In univariate analysis, grade I tumors were associated with a lower risk of mortality (HR = 0.46, 95% CI: 0.22–0.931, P = 0.031). This association remained significant in the multivariate model (HR = 0.46, 95% CI: 0.23–0.97, P = 0.040) (Table 3). These results indicate that higher tumor grades are associated with an increased risk of mortality in patients with NSCLC. Other variables, including EGFR mutation status, tumor histology, gender, and age, did not show statistically significant associations with survival outcomes (P > 0.05).

**Table 3:** Cox Regression Analysis Results for Factors Affecting Patient Survival

Variables	Univariate analysis		Multivariate analysis	
	HR (95%CI)	P-Value	HR (95%CI)	P-Value
EGFR (21 L858R mutation)	0.91 (0.54-1.54)	0.730	-	-
Tumor Type	1.12 (0.94-1.35)	0.197	1.15 (0.93-1.34)	0.730
Tumor Grade 1	0.46 (0.22-0.931)	**0.031**	0.46 (0.23-0.97)	**0.040**
Tumor Grade 2	1.02 (0.80-1.30)	0.880	-	-
Gender	0.91 (0.71-1.16)	0.433	-	-
Age	1.01 (0.99-1.02)	0.614	-	-

## Discussion

This study investigated the relationship between the EGFR exon 21 L858R mutation and clinicopathological characteristics of patients with NSCLC. The average age of the patients in our cohort was 62.96 ± 6.85 years, suggesting that NSCLC predominantly affects older individuals. This observation aligns with findings from a previous study by Mohammadi et al. in Iran, who reported a mean age of 60.5 years in patients with lung adenocarcinoma (25). Similar trends have also been reported globally, including in the study by Wang et al. (26) . These findings highlight the importance of active monitoring and early diagnosis of lung cancer in the elderly, who may be at increased risk due to various factors, such as smoking, environmental exposure, and age-related genetic alterations. 

On the contrary, some studies have shown a higher prevalence of adenocarcinoma among younger patients, while squamous cell carcinoma trends to be more prevalent in older males (27, 28). However, other investigations have not found significant age-related in tumor type prevalence (27).

Another key observation from this study was the lack of a significant relationship between gender and EGFR mutation status. These supports findings from Calibasi-Kocal et al., who reported a similar distribution of EGFR mutations between males and females (29). In addition, Guo et al. suggested that genetic and environmental factors likely play a more decisive role in EGFR mutation than gender alone (30). 

In our cohort, the exon 21 L858R mutation was identified in only 6% of patients, supporting previous reports that have consistently shown a lower prevalence of exon 21 mutations compared to other EGFR mutations, particularly exon 19 deletions, in NCLC patients. For example, Salmani et al. and Batra et al. both found higher frequencies of exon 19 deletions than L858R mutations, especially among Asian populations (31)^,^(32). These mutations, particularly the L858R variant, are known to influence treatment response, especially to tyrosine kinase inhibitors (TKIs), and may offer prognostic implications. However, as supported by our findings and other studies the prognostic impact of L858R remains controversial (20, 33). Recent studies, including subgroup analyses of clinical trials such as FLAURA, have shown that patients with L858R mutations may have slightly reduced response and survival benefit from third-generation EGFR-TKIs like osimertinib compared to those with exon 19 deletions. This emerging evidence highlights the need for more mutation-specific treatment approaches and further investigation into the biological behavior and treatment sensitivity of different EGFR mutation subtypes (34-37).

In term of metastasis, our results showed no significant association between the presence of the L858R mutation and metastatic disease (P = 0.900). This align with some literature suggesting that EGFR mutations do not uniformly predict metastatic potential and depend on the type of mutation and the cellular environment (38-40). For instance, Sharma et al. proposed that EGFR mutations, especially L858R, may be associated with less aggressive tumor behavior in some settings (38). However, other studies have reported that L858R mutations are linked to an increased risk of brain metastases and earlier progression during TKI therapy (41)^,^(42, 43). This discrepancy may be partly explained by co-occurring mutations in tumor suppressor genes, which are more frequently observed in tumors harboring the L858R variant (42). 

Similarly, survival analysis in our study demonstrated no statistically significant difference in overall survival between mutation-positive and mutation-negative patients (P = 0.246). These findings suggest that the prognostic role of the L858R mutation may be context-dependent and influenced by multiple factors, including patient demographics, treatment modalities, and molecular tumor profiles. Our Cox regression model identified tumor grade as the only significant prognostic factor, with higher-grade tumors associated with poorer outcomes. This supports the well-established notion that tumor differentiation is a key determinant of prognosis in NSCLC.

It should be noted that the relatively small number of mutation-positive cases (n=20) in our study limits the statistical power to detect subtle differences in subgroup analyses. Therefore, some null findings, particularly those related to gender, age, tumor grade, metastasis, and survival, may be affected by this limitation, and caution is warranted in interpreting these results. Another important limitation of this study is the lack of comprehensive genomic profiling to detect potential co-occurring mutations, such as TP53, which are increasingly recognized as critical modifiers of both prognosis and response to EGFR-TKIs. Given the complexity and conflicting evidence regarding the prognostic value of the L858R mutation, further large-scale studies with comprehensive molecular profiling are necessary to clarify its role in NSCLC pathogenesis and treatment response.

## Conclusion

In conclusion, exon 21 L858R mutations were detected in only 6% of NSCLC patients in our study, indicating their relatively low prevalence. No statistically significant associations were found between this mutation and key clinical variables such as age, gender, tumor grade, metastasis, or mortality. Given the ethnic and geographic variability in EGFR mutation patterns, our findings provide region-specific insights that differ from both East Asian and Western populations. These differences highlight the necessity for tailored molecular testing protocols in Iran to ensure precise detection of actionable mutations and the implementation of personalized treatment strategies. Future large-scale studies with comprehensive genomic profiling, including assessment of co-occurring mutations, are essential to clarify the biological behavior and therapeutic implications of the L858R mutation and to optimize patient management in the Iranian NSCLC population.

## Authors' Contributors

Study concept and design: Ayatollahi H, & Jafarian AH, Acquisition of data: Oudi B, Emamdadi Z, Ranjbar F, Statistical analysis: Mehrad-Majd H, Drafting of the manuscript: Oudi B, Mehrad-Majd H, Study supervision: Ayatollahi H,

## Data Availability

There is no additional data separate from available in cited references.
